# Supplementary material for: Targeting primary and metastatic uveal melanoma with a G protein inhibitor
Source: J Biol Chem. 2021 Feb 10;296:100403. doi: 10.1016/j.jbc.2021.100403 (PMC7948511; doi:10.1016/j.jbc.2021.100403)
Supplement: Figures S1–S4 and Tables S1–S2 [file mmc2.pdf]

# Supplementary Data

**Table S1:** Uveal melanoma tumor class and genotypes of patient biopsies analyzed by RNAseq

| | Molecular Class | GNAQ | GNA11 |
|---|---|---|---|
| UM030 | 1 | Q209P | wt |
| UM031 | 2 | Q209L | wt |
| UM032 | 1 | wt | wt |
| UM033 | 1 | Q209L | wt |
| MUM04 | n.d. | Q209L | wt |
| UM043 | 1 | wt | Q209L |
| UM044 | 2 | wt | Q209L |
| UM045 | 1 | wt | Q209L |
| UM046 | 2 | wt | Q209L |
| UM047 | 1 | Q209P | wt |
| UM049E | 2 | wt | Q209L |
| UM051E | 2 | wt | Q209L |
| UM052E | 2 | wt | Q209P |

**Supplementary Table S1.** Tumor genotypes of UM patient biopsy samples analyzed by RNAseq. Ten fine-needle aspiration biopsy samples and three enucleation samples were collected from UM patients as described in Experimental Procedures. Molecular classes were determined by Castle Biosciences. GNAQ and GNA11 genotypes were determined from RNAseq data. Molecular classification was not determined for the liver metastasis sample MUM04.

**Table S2:** Effect of systemically administered FR on hematopoiesis

| | vehicle | FR (0.1 mg/kg) | FR (0.3 mg/kg) |
|---|---|---|---|
| WBC ($10^3$ cells/μL) | 2.33±0.57 | 3.27±1.22 | 2.4±0.77 |
| RBC ($10^6$ cells/μL) | 6.72±0.3 | 5.99±0.39 | 6.1±0.42 |
| HGB (g/dL) | 10.11±0.41 | 9.11±0.47 | 9.43±0.44 |
| HCT (%) | 30.8±2.6 | 35.6±1.8 | 34.3±2.1 |
| MCV (fL) | 52.6±1 | 54.1±0.8 | 57±1.2 |
| MCH (pg) | 15.1±0.2 | 15.3±0.3 | 15.6±0.4 |
| MCHC (g/dL) | 28.8±0.6 | 28.4±0.3 | 27.4±0.5 |
| PLAT ($10^3$ cells/μL) | 625±38 | 658±87 | 854±54 |

**Supplementary Table S2.** Effect of FR on hematopoiesis. Blood samples were collected from mice after 30 d of treatment with vehicle or FR (s.c. on alternate days; n=10 mice per group) and assayed for blood cell counts (WBC, white blood cells; RBC, red blood cells; HGB, hemoglobin; HCT, hematocrit; MCV, mean corpuscular volume; MCH, mean corpuscular hemoglobin; MCHC; mean corpuscular hemoglobin concentration; PLAT, platelets). Values shown are mean ± standard error of the mean. No significant changes were seen with FR treatment (t-test).

## Fig. S1

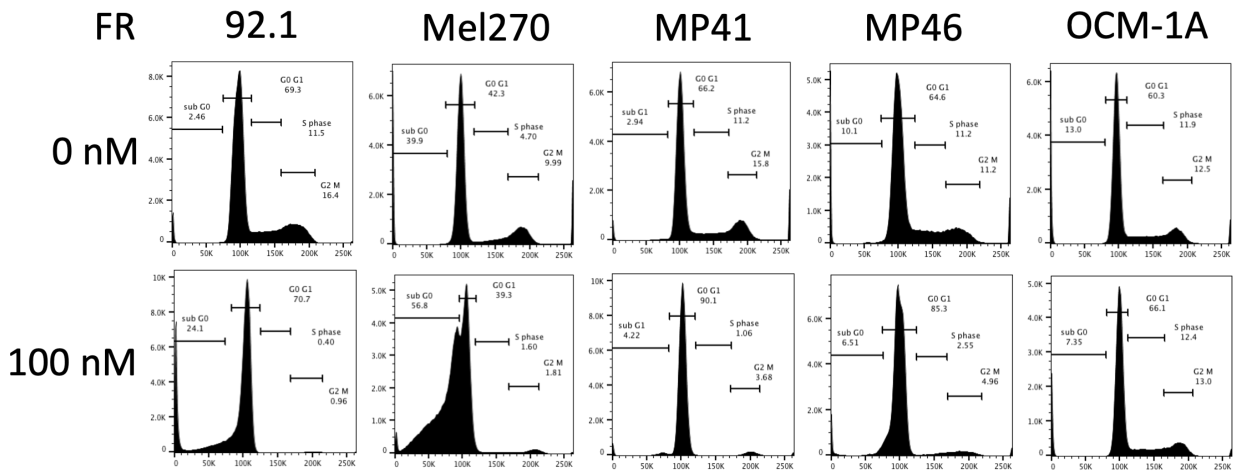


**Supplementary Figure S1.** Effect of FR on cell cycle progression and apoptosis of UM cell lines. Shown are flow cytometric analysis of vehicle- and FR-treated UM cell lines. Representative graphs showing number of cells (y-axis) versus intensity of propidium iodide staining for DNA content (x-axis) for 92.1 (GqQ209L), Mel270 (GqQ209P), MP41 (G11Q209L), MP46 (GqQ209L:BAP1null), and OCM-1A (BRAFV600E) UM cell lines. Top row – vehicle treated; bottom row – FR treated. Gating for cell cycle phases was done on vehicle treated histograms and applied to FR-treated histograms.

Fig. S2

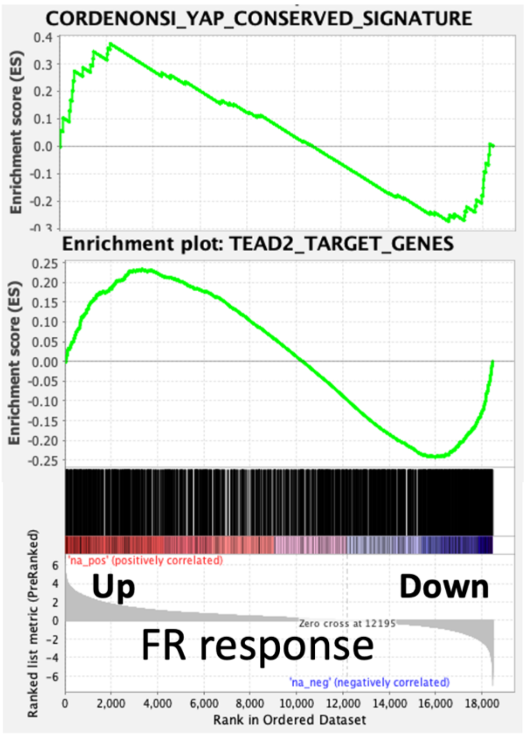


**Supplementary Figure S2.** Gene set enrichment analysis of the YAP pathway in FR treated patient samples. Gene set enrichment analysis for YAP and TEAD target genes was performed using bulk RNAseq data from all FR-responsive human tumor samples. Upregulated and downregulated genes were enriched equally.

# Fig. S3

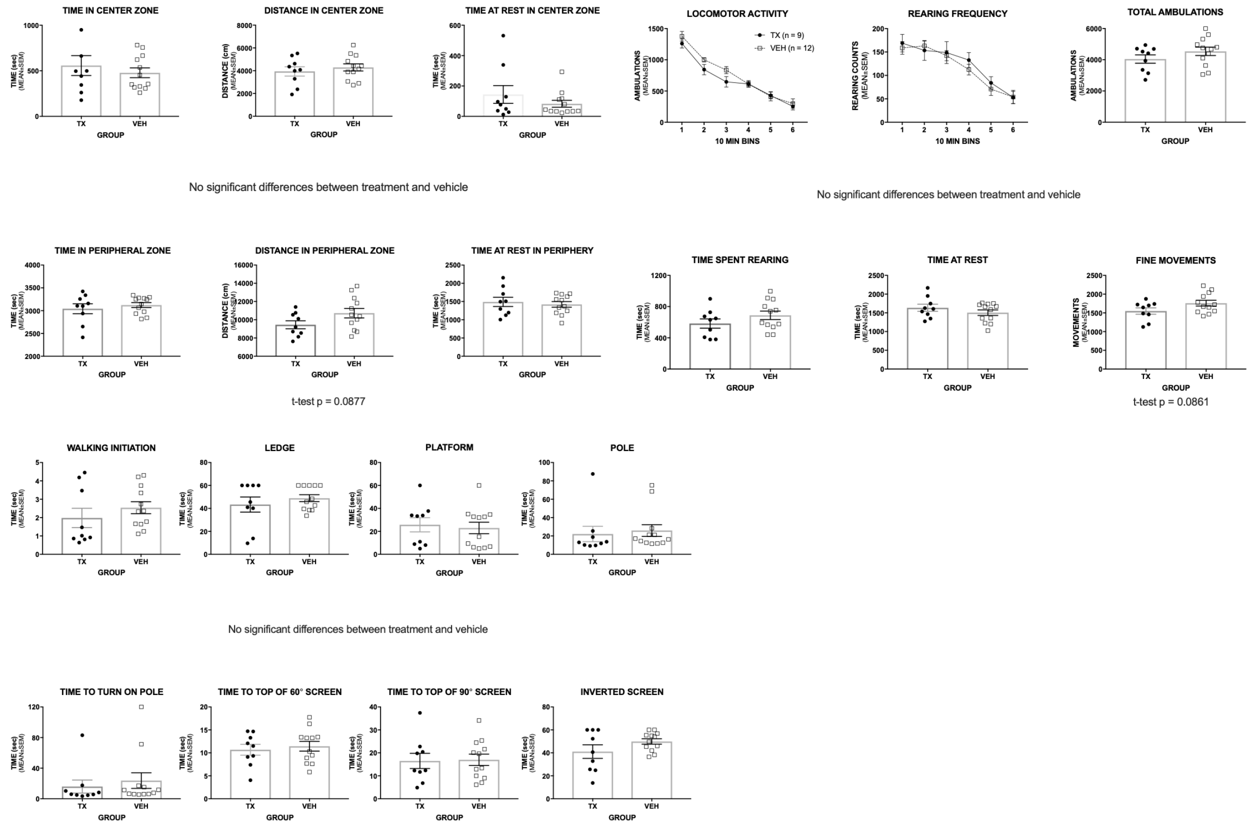


**Supplementary Figure S3.** Mouse behavior in response to FR. Mice (n=12 group) were treated one week by s.c. injection of vehicle or FR (0.3mg/kg on alternate days), and analyzed one day after the last injection. Graphs correspond to data summarized in Table 1. General activity levels and exploratory behavior were quantified over a 60-min period in an open-field, and general-activity variables (total ambulations; rearings; time at rest) along with measures of emotionality, including time spent, distance traveled, and entries made into the central zone were analyzed. Time in walking initiation, ledge, platform, pole, and inclined and inverted screen tests were manually recorded. The average of two trials was used for each analysis. No significant differences ($p < 0.05$) were detected between control and FR-treated groups.

## Fig. S4

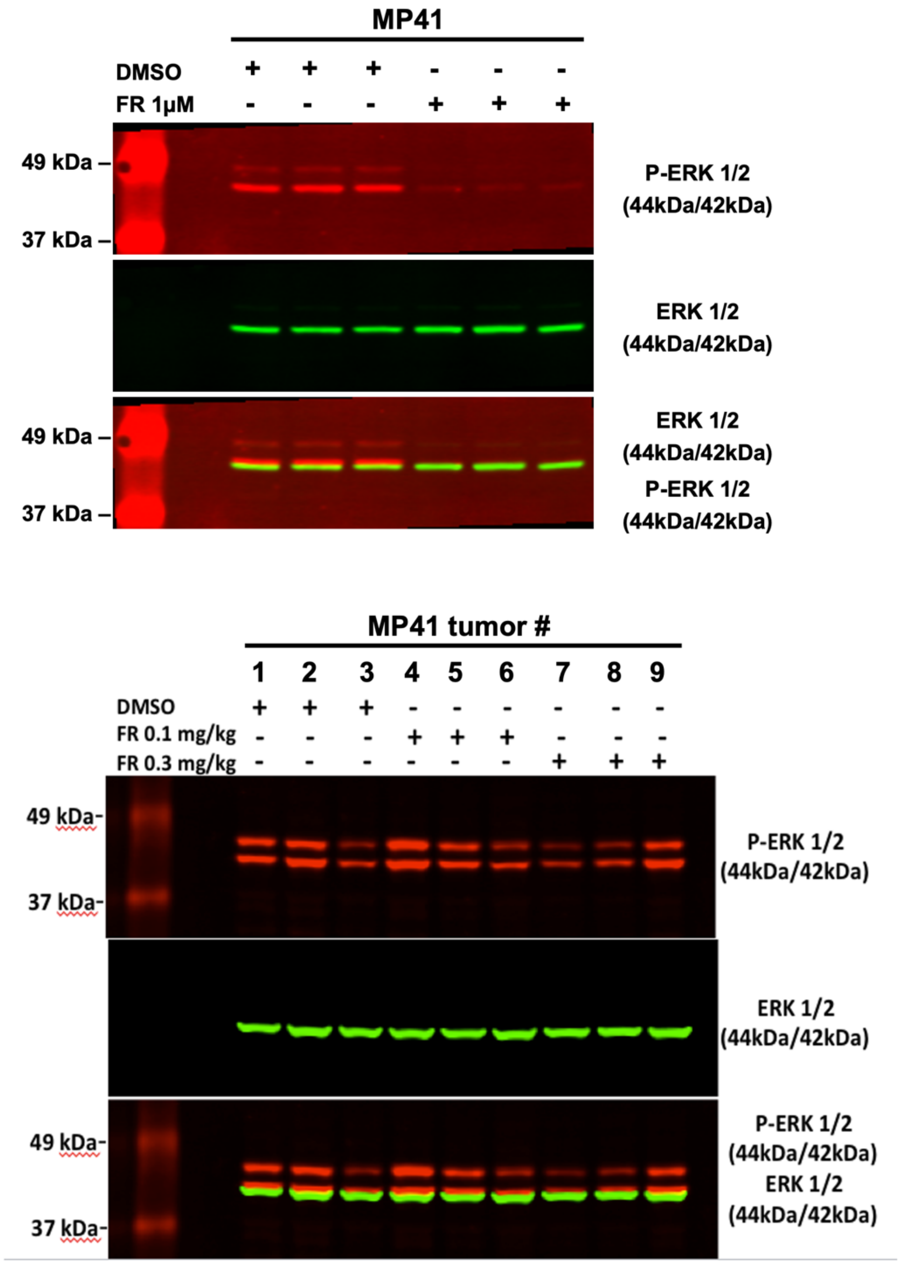


**Supplementary Figure S4.** Effect of FR on phospho-ERK and total ERK in cultured UM cells and xenografted UM tumors. Cultured UM cells were treated with vehicle (DMSO) or 1 µM FR for 18 hr. Approximately 100 mg of xenografted tumor was collected from vehicle- and FR-treated mice (n=9 mice/condition). Immunoblots were probed with primary antibodies against phospho-p44/42 MAPK (P-ERK1/2) and p44/42 MAPK (ERK1/2) followed by IRDye–coupled

secondary antibodies. Signals were detected using Odyssey model 9120 imaging system (LI-COR Biosciences). Panels show phospho-ERK1/2 (top), total ERK1/2 (middle) and merged fluorescence signals (bottom) from a single experiment in which each lane shows the result obtained under each condition from an independent cultured-cell sample or tumor. Results shown are representative of three independent experiments.

**Supplementary Data File (.xlsx)** List of genes showing significant response to FR as detected by RNAseq. ENSEMBL identifiers, Genbank names and brief descriptions are given for each gene. Data in columns are log2 fold change and p-value (two-tailed, paired T-test) based on bulk RNAseq cpm data for all nine FR-responsive samples or broken out into Class 1 and Class 2 sample subsets.
